# Clinical significance of Akt and HER2/neu overexpression in African-American and Latina women with breast cancer

**DOI:** 10.1186/bcr1844

**Published:** 2008-01-10

**Authors:** Yanyuan Wu, Hezla Mohamed, Ram Chillar, Ishrat Ali, Sheila Clayton, Dennis Slamon, Jaydutt V Vadgama

**Affiliations:** 1 Divisions of Cancer Research and Training, Hematology/Oncology, Department of Medicine; 2 Charles R. Drew University of Medicine and Science, 1731 East 120^th ^Street, Los Angeles, CA 90059, USA; 3 David Geffen UCLA School of Medicine, Los Angeles, CA, USA; 4 Department of Pathology, Charles R. Drew University of Medicine and Science, 1731 East 120^th ^Street, Los Angeles, CA 90059, USA; 5 Department of Surgery, Charles R. Drew University of Medicine and Science, 1731 East 120^th ^Street, Los Angeles, CA 90059, USA; 6 Division of Hematology/Oncology, Department of Medicine,10833 Le Conte Avenue, Los Angeles, CA 90095

## Abstract

**Introduction:**

Breast cancer patients with HER2/neu overexpression have poor outcomes with a decrease in disease-free survival (DFS) and overall survival. The biology of HER2/neu overexpression in breast tumors in African-American and Latina women is poorly understood. The purpose of this study is to understand the clinical significance of activated Akt (phospho-Akt or pAkt) expression in breast tumors from African-American and Latina patients with corresponding tissue HER2/neu overexpression. Cellular and molecular studies have shown that activation of the cell signaling phosphatidylinositol-3-kinase/Akt cascade via the HER2/neu and other receptor tyrosine kinases induces cell proliferation.

**Methods:**

A total of 234 African-American and Latina patients were selected retrospectively. From this group, 141 tumor tissue samples were analyzed for tissue pAkt by immunohistochemistry (IHC). This cohort consisted of 46 HER2/neu-positive (3+ by IHC) and 95 HER2/neu-negative tumors. The prognostic value of activated tissue Akt in relation to HER2/neu overexpression for DFS was determined.

**Results:**

Patients with low pAkt and HER2-negative tumors had the best DFS. As expected, HER2/neu-overexpressing tumors with low pAkt had a decrease in DFS. Similarly, those with high pAkt and HER2-negative tumors also had poor DFS. However, those with an increase in both HER2 and pAkt had the worst DFS. An increase in pAkt was significantly associated with HER2/neu-positive and lymph node-positive breast tumors. Tumors with high HER2 and high pAkt were metastatic. Multivariate analysis demonstrated that, in addition to the common risk factors such as larger tumor size, lymph node involvement, estrogen receptor/progesterone receptor-negative tumors, and HER2/neu-positive tumors, overexpression of pAkt significantly was associated with a decrease in 5-year DFS. A decrease in DFS with an increase in pAkt was observed in both HER2/neu-positive and -negative groups. However, the DFS was similar between HER2/neu-positive/pAkt-negative and HER2/neu-negative/pAkt-positive groups.

**Conclusion:**

Our data suggest that there may be differences in tumor phenotypes within the HER2/neu-overexpressing breast cancer patients. The overexpression of pAkt may be a powerful prognostic marker for predicting DFS and overall survival of breast cancer patients.

## Introduction

Breast cancer is the most common non-dermatologic cancer among American women and ranks second among cancer deaths in women. Although breast cancer survival has improved over the last 30 years, unexplained cancer-related health disparities still remain between African-Americans and Caucasians. People with low socioeconomic status still have the highest rates of both new tumors and cancer deaths. African-Americans are at a higher risk to die from cancer than are other ethnic groups [1]. Several factors have been demonstrated to contribute to poor outcome in African-American women. Most often, late stage at diagnosis has been a significant contributor. Breast cancer also is the most commonly diagnosed cancer and the leading cause of cancer death among Hispanic/Latina (Latina) women, even though breast cancer incidence and mortality in Latina women were not as high as those of African-American and Caucasian women. Despite recent increases in screening rates, breast cancer still tends to be diagnosed at a later stage in many Latina women, when treatment options are more limited. Uninsured Latina women are two to three times more likely to be diagnosed at a later stage [2]. Our medical center is located in South Central Los Angeles and serves primarily underserved populations, mainly African-American and Latina patients. Eighty percent of these African-American and Latina women had no health insurance. Earlier studies from our laboratory have shown that the age of onset of breast cancer for Latina women is younger than for African-Americans (48 versus 53 years) and that both of them have advanced stage III/IV disease at the time of diagnosis and had poor disease outcome [3].

In general, the association between poor survival and differences with tumor phenotypes is not well understood in minority women with breast cancer. There is a need to develop novel or better therapeutic strategies in the management of breast cancer in African-American and Latina women. Significant research efforts are ongoing to better understanding the molecular basis of breast cancer and the discovery of molecular markers that could predict reliable prognostic or treatment outcome [4-7]. Experimental studies have suggested that overexpression of different growth factor receptors in breast cancer mediates different cell signaling pathways and makes the cancer cells become less responsive to treatment. These receptors include insulin-like growth factor receptor and members of the epidermal growth factor receptor (EGFR) family, such as HER1 (EGFR) and HER2 [8-11].

HER2/neu (also called c-erbB2), a cell-surface membrane receptor, has been identified and recognized to be significantly associated with breast cancer recurrence and death in the last two decades. In general, about 20% to 30% of breast cancer patients are diagnosed with an aggressive form of cancer that is associated with overexpression of the HER2/neu protein and its gene. HER2/neu-overexpressing tumors frequently become resistant to treatment with tamoxifen and/or chemotherapy [12-15]. Current treatment regimens combining trastuzumab (Herceptin) with paclitaxel and/or docetaxel [16,17] have shown increased response rate. However, greater than 70% of patients with HER2/neu-overexpressing tumors show poor response to treatment [18,19]; in these patients, the overall survival (OS) and the time to relapse are significantly shorter [17].

Several mechanisms that explain the mode of resistance to therapy by the chemo agents alone or in combination with trastuzumab have been described [11,20-22]. The phosphatidylinositol-3-kinase (PI3K) and its associated protein kinase B (Akt) pathways are frequently activated upon stimulation of various receptor-mediated cellular signals [23-25]. The activation of this PI3K/Akt signaling in response to growth factor receptor activation leads to anti-apoptosis and pathogenesis of cancer [26]. The PI3K/Akt pathway can be activated by several growth factors: insulin-like growth factor, epidermal growth factor, cytokines, and the HER2/neu network. The activation of Akt results in the downstream regulation of target molecules: glycogen synthase kinase-3-beta [27], Forkhead transcription factor [28], caspase-9 [29], and pro-apoptotic Bcl-2 family member Bad [30]. The final outcome may result in cellular proliferation or anti-apoptosis [31,32].

Recent studies using clinical specimens have shown that Akt protein is frequently activated in HER2/neu-overexpressing breast tumors and is associated with poor prognosis among tamoxifen-treated patients [33-37]. Studies on Akt status in relation to prognosis of breast cancer were mostly focused on patients with estrogen receptor (ER)-positive tumors treated with hormone therapy [35,36]. There is limited information on Akt status in patients with ER-negative tumors and the predictive value of Akt on those breast cancer patients. In addition, most of these studies were done in either Caucasian or Asian patients [34-36]. In contrast, there is a dearth of studies on clinical outcome in African-American and Latina patients with tissue HER2/neu overexpression with a corresponding increase in activated Akt at the time of initial diagnosis. From our early studies, we have observed that an increased level of plasma HER2/neu in both African-American and Latina women with breast cancer who underwent surgery and completed chemotherapy was associated with poor outcome and a reduction in disease-free survival (DFS) [3].

In the present study, we hypothesized that a subgroup of patients with HER2/neu-overexpressing breast cancer will also demonstrate an increase in activated Akt in their tumors. These patients will be resistant to chemotherapy and consequently this group of patients will have a shorter DFS and a poor OS. The activation of Akt in breast tumors could be a potential biologic factor that may partially explain the worse outcome in those minority women with breast cancer. Hence, our primary goals were (a) to test whether pAkt overexpression in HER2/neu-overexpressing tumors led to poor outcome compared with HER2/neu-overexpressing tumors but with normal or low levels of pAkt, (b) to compare them to levels of pAkt in HER2/neu-negative tumors, and (c) to focus on African-American and Latina patients at our medical center, where these two populations have similar socioeconomic status and access to care. We have postulated that the overexpression of pAkt will lead to poor outcome irrespective of ethnic or racial differences. We studied a total of 141 patients. The 5-year DFS and the relative risk (RR) of reducing 5-year DFS were estimated in these minority patients with HER2/neu-positive and -negative and with or without pAkt in their tumors. Finally, we have correlated pAkt levels according to the tumor subtypes and we determined DFS rates.

## Materials and methods

### Patients

In this study, we were unable to include Caucasians. This is primarily due the fact that the majority of our patient populations at our medical center in South Central Los Angeles are poor and underserved African-American and Latina patients. The Caucasian population is less than 2.0% and therefore we are unable to generate a meaningful sample size.

Patients included in this study were self-reported as African-American and Latina women with breast cancer and had undergone surgery. They were treated with chemo or adjuvant chemotherapy at King/Drew Medical Center between 1999 and 2005. Approval from the institution review board was obtained before initiating the study. Presurgical information, tumor pathology and histology (that is, TNM [tumor, node, metastasis] staging, HER2/neu status, and ER or progesterone receptor [PR] status), and treatment protocol as well as disease outcome were compiled on patients from our prospective database. The ER/PR status and HER2/neu status reported in patient pathology reports were determined by immunohistochemistry (IHC) and provided by Impath, the Cancer Information Company (Los Angeles, CA, USA). ER/PR-positive status was considered as more than 5% staining in cell nuclei; otherwise, ER and/or PR status was considered as negative. The expression of HER2/neu protein determined by IHC was also obtained according to the pathologist's interpretation of the IHC stain. The IHC stain was performed by the same central laboratory that performed the ER/PR assays. The IHC was performed with the DAKO HERCEPTest [38] and scored on a qualitative scale from 0 to 3+. The staining intensities are defined as 3+ positive, which means a strong complete membrane staining in more than 10% of tumor cells; 2+ positive, which is a weak to moderate complete membrane staining in more than 10% of tumor cells; 1+ positive, which is faint or barely perceptible partial membrane staining in more than 10% of tumor cells; and 0 positive, no staining is observed or membrane staining is observed in less than 10% of tumor cells. The HER2/neu status was defined as positive (HER2/neu 3+ determined by IHC) or negative (included HER2/neu 2+ and 1+/negative determined by IHC) in this study.

Patients included in this study were also required to have pathologically confirmed ductal or lobular breast carcinoma. We excluded those whose breast cancer was not primary cancer, had no detailed information on their tumor pathology, and had not completed treatment protocol for other than clinical reasons. Each patient included in this study had given their informed consent.

We selected cases (with paraffin blocks) that had known corresponding HER2/neu information and whose paraffin slides for tumor tissue contained more than 10% tumor cells. A total of 141 patients fulfilled our criteria and were retrospectively selected. The number of African-American patients was 72 and the number of Latina patients was 69. In this cohort of 141 breast cancer patients, 113 had invasive ductal (106) or lobular (7) carcinoma and 28 of them had ductal carcinoma *in situ *(26) or lobular carcinoma *in situ *(2). We had follow-up information on all 141 patients.

### Determination of pAkt expression by immunohistochemistry

To perform IHC on human paraffin tissue samples, 4-μ sections were placed onto poly-prep slides (P0425; Sigma-Aldrich, St. Louis, MO, USA) and dried at 60°C for 1 hour. The sections were deparaffinized and hydrated in 100% ethanol followed by 95% ethanol. For antigen retrieval, paraffin tissue sections were treated with sodium citrate (10 mM, pH 6.0) for 10 minutes at 95°C and then cooled for 30 minutes at room temperature. Immunostaining was performed by using a Vectastain Universal *elite *ABC kit (PK-6200; Vector Laboratories, Burlingame, CA, USA). Each tissue section was blocked with 5% normal horse serum for 30 minutes followed by overnight incubation at 4°C with antibodies specific for phosphor-Akt (Ser473) (pAkt) (#9271; Cell Signaling Technology, Inc., Danvers, MA, USA) and total Akt (#9272; Cell Signaling Technology, Inc.). Immunostaining was visualized with a streptavidin peroxidase reaction using the DAB (3,3'-diaminobenzidine) kit (SK-4100; Vector Laboratories). The nuclei were counterstained with hematoxylin before mounting. Negative-control tests were conducted with samples in the absence of primary antibody. Similarly, control paraffin slides with known negative or positive expression of pAkt (IHC confirmed and antibody supplied by vendor) were tested alongside the unknown samples.

**Figure 1:**
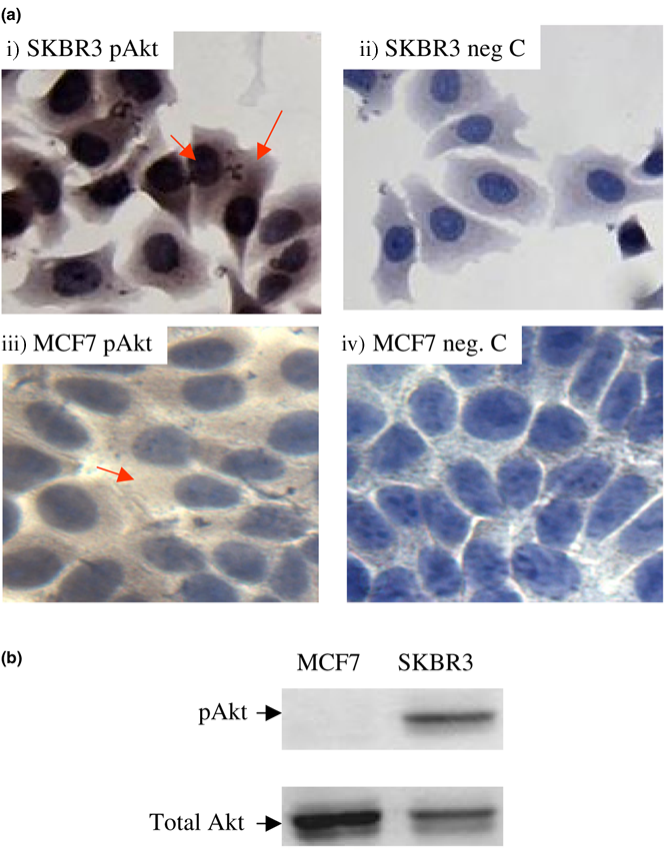
pAkt expression in human breast cancer cells determined by immunohistochemistry and Western blot analysis. **(a) **Immunohistochemistry of pAkt expression in SKBR3 and MCF7 cells: (i) SKBR3 cells stained with pAkt (Ser473) antibody, (ii) SKBR3 cells stained with secondary antibody only (negative control), (iii) MCF7 cells stained with pAkt (Ser473) antibody, and (iv) MCF7 cells stained with secondary antibody only. The arrows indicate very strong pAkt stain in the cytoplasm and nuclear areas of SKBR3 cells, whereas light pAkt stain was observed in the cytoplasm of MCF7 cells. **(b) **Western blot analysis on total protein from SKBR3 or MCF7 cells using antibodies directed against pAkt (Ser473) (top panel) and total Akt (bottom panel).

**Figure 2:**
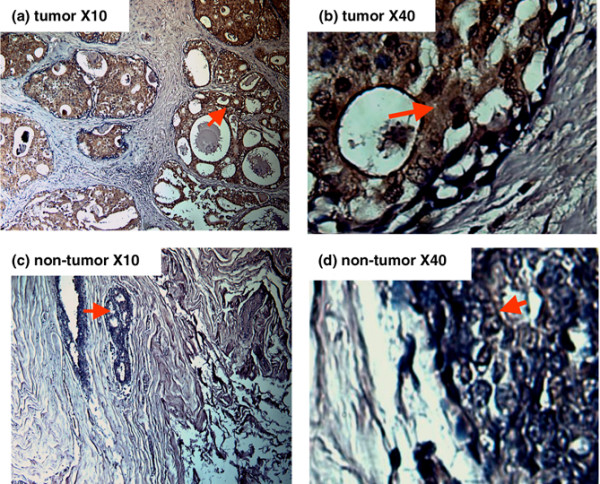
pAkt expression in tumor and uninvolved tumor tissue from the same patient examined by immunohistochemistry (IHC). Low-power **(a) **and high-power **(b) **images from the same tissue section contained *in situ *and invasive ductal carcinoma and had clear IHC stain in the cytoplasm. Low-power **(c) **and high-power **(d) **images from the same tissue section without tumor had no or light staining.

Before testing the paraffin tissues for pAkt by IHC, the sensitivity and specificity of pAkt antibody were tested and validated using human breast cancer cell lines obtained from the American Type Culture Collection (ATCC) (Manassas, VA, USA), such as SKBR3 (overexpressing HER2/neu) and MCF7 (expressing moderate levels of HER2/neu). The pAkt levels in SKBR3 and MCF7 were determined by IHC first and then further confirmed by Western blot analysis (Figure 1). A consistent correlation was observed between IHC and Western blot analysis with respect to the level of pAkt expression in SKBR3 and MCF7 cells. We have also compared the staining level on tumor and non-tumor sections from the same patient. Figure 2 demonstrates a paired tissue section from a patient with moderately differentiated infiltrating duct carcinoma who underwent modified radical mastectomy. Figures 2a and 2b are from a tumor and adjacent biopsy cavity that contained *in situ *and invasive ductal carcinoma and had a clear IHC stain in the cytoplasm. Figures 2c and 2d are from uninvolved breast tissues adjacent to the biopsy cavity and had no or very light stain.

### Quantification of immunohistochemistry results

**Figure 3:**
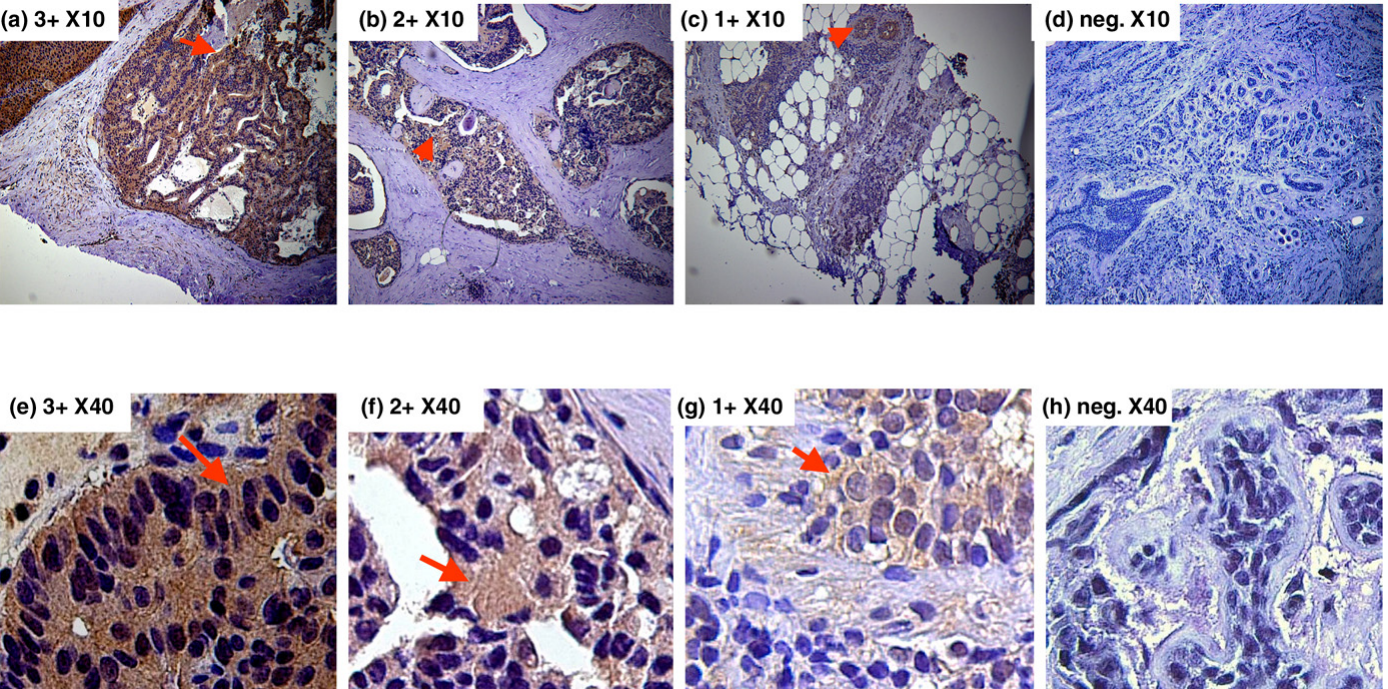
The intensity of immunohistochemistry staining with pAkt in breast tissue. **(a) **3+ positive (intensity was very strong). **(b) **2+ positive (clear stain but the intensity was not as strong as 3+). **(c) **1+ positive (some light stain). **(d) **Negative (no stain). **(a-d) **Low-power images. **(e-h) **High-power images corresponding to **(a-d**), respectively.

**Figure 4:**
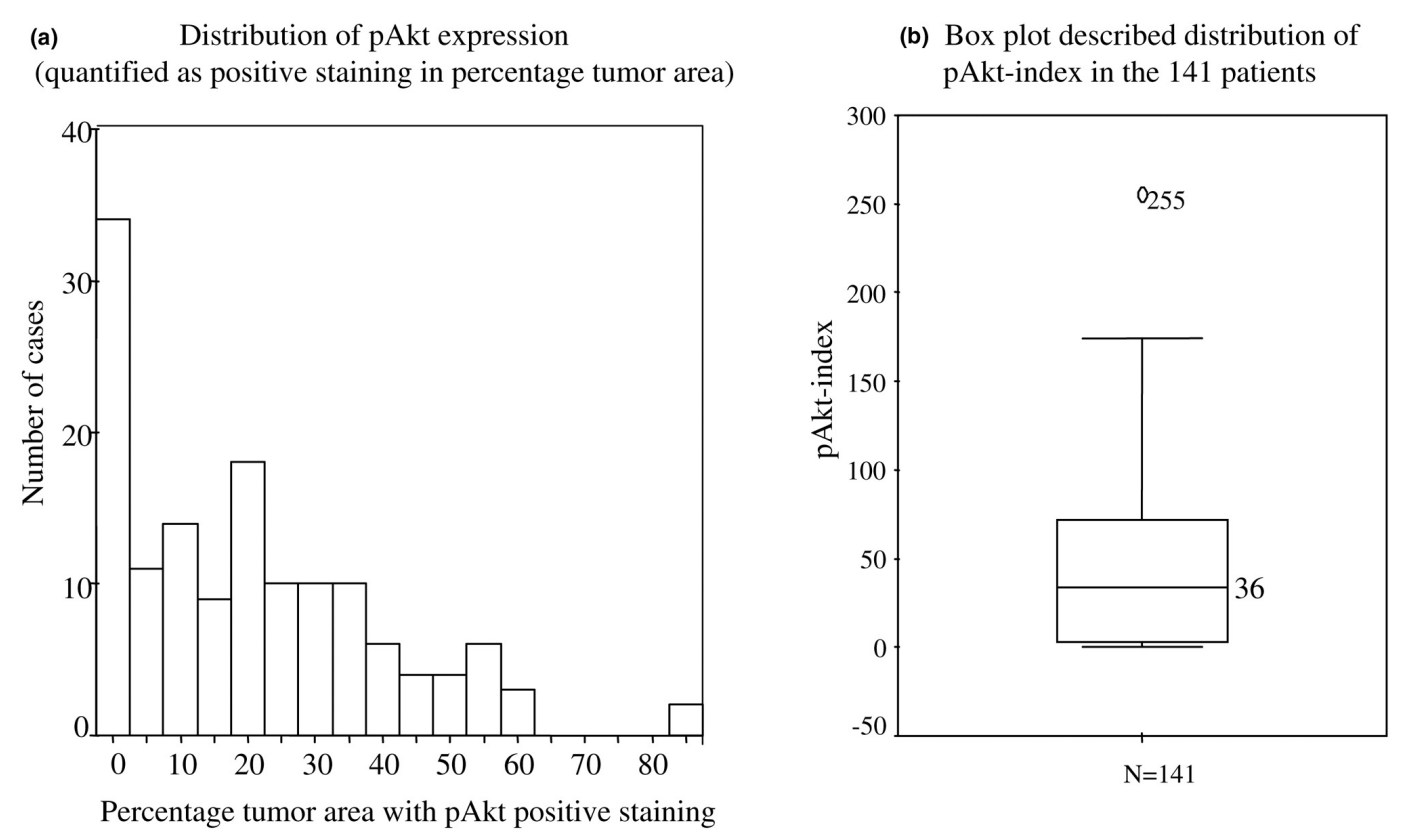
Distribution of pAkt expression. **(a) **pAkt level determined by immunohistochemistry (IHC) was quantified using DigiPro software (Labomed, Inc., Culver City, CA, USA) according to the percentage of tumor area with positive pAkt stain as mentioned in Materials and methods. The x-axis indicates the percentage of tumor area with positive stain. The range of the percentage of tumor area stained positive for pAkt was from 0% (negative stain) to 85% and was plotted at 5% intervals. The y-axis indicates the total number of cases at each of the 5% intervals. Each bar represents the total number of cases within the indicated percentage of tumor area which was positively stained by pAkt. **(b) **The box plot describes the distribution of pAkt index in 141 patients. The level of pAkt determined by IHC and quantified with DigiPro software represents pAkt index as stated in Materials and methods (pAkt index = intensity × percentage of tumor area with positive stain). The range of pAkt index in the 141 patients is described by the box plot. The median level of pAkt index was 36. The value 255 was one outlier.

The pAkt expression was detected in the cytoplasm by IHC. A mixed cytoplasm and nuclear staining was observed in a few cases. The pAkt protein expression was first evaluated using microscopy by two investigators independently without any prior knowledge of clinical information and pathological parameters. The stain color and intensity ranged from light to dark brown. We have scored the intensity of staining as 3+ positive (very strong), 2+ positive (clear staining but not as strong as 3+), 1+ positive (some lighter staining), and negative (no staining) (Figure 3). Next, the pAkt expression was quantified using DigiPro software (Labomed, Inc., Culver City, CA, USA) according to the percentage of tumor area with positive cytoplasm staining (positive staining tumor area divided by total tumor area). Each sample was randomized and quantified from five different areas on the tissue slide, and the mean of five different areas on the slide was calculated. The standard deviation (SD) was less than 2% for each. The distribution of mean area of pAkt-positive expression from those tissue samples was from 0% to 85% (Figure 4a). The quantification of IHC results of pAkt expression using both microscopy for intensity level and computer software for areas was confirmed by the clinical pathologist who was blinded to the origin of the tissue. There were few cases in which the pathologist disagreed with the initial scores. For those cases, we discussed them with the pathologist and then objectively re-scored them and re-confirmed them by two pathologists. In final the analysis, we developed the pAkt index by combining stain intensity in the cytoplasm of tumor cells and percentage of tumor cells with cytoplasm staining (intensity × positive area). The range of pAkt index for the majority of cases was from 0 (negative staining) to 174 (Figure 4b). The median level of the pAkt index was 36, and the pAkt index was 255 in one case only. In the following analysis, the level of pAkt expression was evaluated using the pAkt index either as a continuous variable directly or categorized as high pAkt (above the median level of the pAkt index, >36) and low pAkt (less than or equal to the median level, ≤ 36). The cutoff level was determined after reviewing different studies (Table 1) [33,36,37,39-43] and further validated by examining the non-tumor sections from the same patients in a total of 30 cases. The pAkt levels were either lower or undetectable in those non-tumor tissue sections, but not in tumor tissue sections (Figure 2). Overall, the pAkt index in those normal sections that had some level of IHC staining was 10.1 ± 4.7 (mean ± SD), the median level was 11, and the range was from 4 to 16.

**Table 1:** Scoring pAkt expression determined by immunohistochemistry

Author/Reference	Method and cutoff level	Antibody (company)	Positive rate, type of cancer, (city)
Stål, *et al*. [37]	>10% strong staining in tumor cells	pAkt-Ser473 (Upstate Biotechnology, Lake Placid, NY, USA)	27%, breast cancer, (Stockholm, Sweden)
Kirkegaard, *et al*. [36]	Above median level of histoscore (inter-quartile range)	pAkt-Ser473 (Biosource International, Camarillo, CA, USA)	51%, breast cancer, (Glasgow, UK)
Tokunaga, *et al*. [33]	≥ 10% of cytoplasm staining	pAkt-Ser473 (Cell Signaling Technology, Inc., Danvers, MA, USA)	34%, breast cancer, (Fukuoka, Japan)
Messersmith, *et al*. [39]	Quantify by Automated Cellular Imaging System (ACIS II, Chromavision, Inc). (combination of staining color, density, and darkness) as a continuous variable	pAkt-Ser473 (Cell Signaling Technology, Inc.)	High level in tumor, colorectal cancer, (Maryland, USA)
Bose, *et al*. [40]	Scoring as 0 to 2 according to intensity; 2 (overexpression) means staining more than normal cells	pAkt-Ser473 (Cell Signaling Technology, Inc.)	36%, breast cancer, (Los Angeles, CA, USA)
Massarelli, *et al*. [41]	More than 15% cytoplasm staining	pAkt-Ser473 (Cell Signaling Technology, Inc.)	46%, tongue cancer, (Houston, TX, USA)
Ogino, *et al*. [42]	Tumor cells were scored as negative or positive, using normal epithelial cells and lymphocytes as reference	pAkt-Ser473 (Cell Signaling Technology, Inc.)	14%, colorectal cancer, (Boston, MA, USA)
Schmitz, *et al*. [43]	More than 45% tumor cells staining in nuclei	pAkt-Ser473 (Santa Cruz Biotechnology, Inc., Santa Cruz, CA, USA)	88%, breast cancer, (Essen, Germany)
Our study	Combination of staining intensity and percentage of tumor cells as pAkt index, according to quartiles of pAkt index	pAkt-Ser473 (Cell Signaling Technology, Inc.)	50%, breast cancer, (Los Angeles)

### Statistical analysis

All of the analyses were performed with a statistical package, SPSS (SPSS Inc., Chicago, IL, USA). DFS was defined as the time from the day of diagnosis by tissue biopsy to the development of either local recurrence or distant metastases. Kaplan-Meier survival curves with log-rank testing were used to assess the DFS. The RR of reducing DFS was determined by Cox proportional hazard regression with multivariate analyses. The personal chi-square test was used to examine the statistically significant differences between activation of Akt and other known predictive markers (tumor size, node involvement, staging, grade, ER/PR status, and HER2/neu status). A *p *value of less than 0.05 was considered statistically significant.

### Sample size

We have based the information of patients with HER2/neu status from our ongoing breast studies. The time of DFS in patients with HER2/neu-negative tumors was 4.3 years and in those with HER2/neu 3+ tumors was less than 3 years. To estimate the study power, we used Epi Infor 2000 (Centers for Disease Control and Prevention, Atlanta, GA, USA), and PS-Power and Sample Size Calculation (Department of Biostatistics, Vanderbilt University, Nashville, TN, USA). We expected that patients with HER2/neu-positive and overexpressing pAkt would have a shorter DFS than those with HER2/neu-positive but low pAkt. To have 80% power with 95% confidence, the sample size should be 149. Therefore, the current sample size will provide approximately 80% power with 95% confidence.

## Results

### Evaluation of pAkt expression by immunohistochemistry

The pAkt antibody was tested using human breast cancer cell lines obtained from the ATCC. These were SKBR3 (overexpressing HER2/neu) and MCF7 (expressing low levels of HER2/neu). The pAkt levels in SKBR3 and MCF7 were determined by IHC first and then examined by Western blot analysis (Figure 1). A consistent correlation was observed between IHC and Western blot analysis with respect to the level of pAkt expression in SKBR3 and MCF7 cells. We have also compared the staining level in tumor and non-tumor sections from the same patient. Figure 2 demonstrates paired tissue sections from a patient with moderately differentiated infiltrating duct carcinoma who underwent modified radical mastectomy. Figures 2a and 2b are from a tumor and adjacent biopsy cavity that contained *in situ *and invasive ductal carcinoma and showed clear IHC staining in the cytoplasm. Figures 2c and 2d are from uninvolved breast tissues adjacent to the biopsy cavity and had no or very light stain.

We have evaluated different studies that have used the IHC scoring method for pAkt. The similarities and differences are documented in Table 1. From our study, we determined that both the intensity of the IHC staining of pAkt and the percentage of tumor cells stained with pAkt were necessary determinants for the scoring system. Similar methods were used by other investigators [36,39]. Hence, we developed a pAkt index by combining both intensity of IHC and percentage of tumor cells stained as stated in Materials and methods. To validate our scoring system, we examined non-tumor sections from the same patients in a total of 30 cases. The pAkt levels were either lower or undetectable in those non-tumor tissue sections, but not in tumor tissue sections. Figure 2 demonstrates one example. Overall, the pAkt index in those normal sections that had some level of IHC staining was 10.1 ± 4.7 (mean ± SD), the median level was 11, and the range was from 4 to 16.

### Characteristics of patients and their tumor clinicopathology

Table 2 provides a description of our patients and their tumor characteristics. Overall, from a cohort of 141 patients, 51.1% were African-American and 48.9% were Latina. The difference in the number of subjects in the study was not statistically significant.

**Table 2:** Patient characteristics

	Number	Percentage	*P *value
Total	141	100	

Ethnicity			
African-American	72	51.1	
Latina	69	48.9	0.801
Age at diagnosis			
<50 years	73	51.8	
≥ 50 years	68	48.2	0.674
Tumor size			
<5 cm	90	63.8	
≥ 5 cm	51	36.2	0.001
Lymph node status			
Positive	89	63.1	
Negative	52	36.9	0.004
AJCC stage			
0–II	88	62.4	
III–IV	53	37.6	0.004
Histological grade			
Well to moderately differentiated	43	30.5	
Poorly differentiated	98	69.5	0.001
ER/PR status			
ER/PR^+^	81	57.4	
ER/PR^-^	60	42.6	0.086
HER2/neu status			
Positive	46	32.6	
Negative	95	67.4	0.001
pAkt level			
High	71	50.4	
Low	70	49.6	0.933

AJCC, American Joint Committee on Cancer; ER/PR, estrogen receptor/progesterone receptor; HER2/neu negative, HER2/neu 2+ and 1+ and negative; HER2/neu positive, HER2/neu 3+ determined by immunohistochemistry; pAkt level high, pAkt index of greater than 36 (median level); pAkt level low, pAkt index of less than or equal to 36.

### Age

Fifty-one point eight percent of our subjects were under 50 years of age, whereas 48.2% were above 50 years of age. There was significant (*p *= 0.001 to 0.004) difference with the following parameters: tumor size, lymph node status, American Joint Committee on Cancer (AJCC) stage, histological grade, and HER2/neu status. A larger number of tumors were less than 5 cm, whereas a greater number had positive lymph node metastasis (63.1%), AJCC stage between 0 and II (62.4%), and poorly differentiated tumors (69.5%), and 32.6% had HER2/neu-positive tumors. Fifty-seven point four percent had ER/PR^+ ^status whereas 42.6% had ER/PR^- ^status. This difference was not significant (*p *= 0.086). Overall, there was no significant difference between the numbers of patients with high pAkt (50.4%) compared to those with low pAkt (49.6%). The pAkt level is measured as pAkt index, and the cutoff value was 36. Please see Materials and methods for details on the estimation method for the pAkt index.

### pAkt expression in relation to patient characteristics and tumor pathology

Next, we examined whether the pAkt levels could be correlated with patient characteristics and tumor pathology (Table 3). Overall, there was no statistical difference in the pAkt levels in tumor tissues between African-American and Latina patients. Similarly, there was no statistical difference in the pAkt levels between patients under or above 50 years of age, with tumors smaller or larger than 5 cm, between AJCC stage below or above stage II, and those with ER/PR^+ ^or ER/PR^- ^tumors. In contrast, pAkt levels were significantly different in tumor tissues obtained from patients with lymph node metastasis and those with HER2/neu-positive tumors. Patients with poorly differentiated tumors also had higher pAkt levels, although the difference was not highly significant (*p *= 0.094). These data suggest that pAkt is elevated significantly only in tumor tissues from patients with lymph node metastasis and HER2/neu overexpression. We next examined the relationship between HER2/neu overexpression, pAkt overexpression, and 5-year DFS.

**Table 3:** pAkt expression in relation to patient characteristics and tumor pathology

	Number	pAkt		*P *value
		Median	Range	

Total	141	36.0	0–255.0	
Ethnicity				
African-American	72	35.7	0–174.0	
Latina	69	36.6	0–255.0	0.992
Age at diagnosis				
<50 years	73	31.0	0–255.0	
≥ 50 years	68	39.0	0–174.0	0.678
Tumor size				
<5 cm	90	32.2	0–174.0	
≥ 5 cm	51	36.6	0–255.0	0.117
Lymph node status				
Positive	89	45.0	0–255.0	
Negative	52	27.5	0–135.0	0.001
AJCC stage				
0–II	88	33.3	0–174.0	
III–IV	53	42.0	0–255.0	0.160
Histological grade				
Well to moderately differentiated	44	30.0	0–174.0	
Poorly differentiated	97	40.8	0–255.0	0.094
ER/PR status				
ER/PR^+^	81	30.0	0–255.0	
ER/PR^-^	60	47.3	0–170.0	0.164
HER2/neu status (IHC)				
Positive	46	44.5	0–255.0	
Negative	95	30.0	0–168.9	0.009

AJCC, American Joint Committee on Cancer; ER/PR, estrogen receptor/progesterone receptor; HER2/neu negative, HER2/neu 2+ and 1+ and negative determined by immunohistochemistry (IHC); HER2/neu positive, HER2/neu 3+ determined by IHC.

### Five-year disease-free survival in relation to HER2/neu and pAkt status

**Figure 5:**
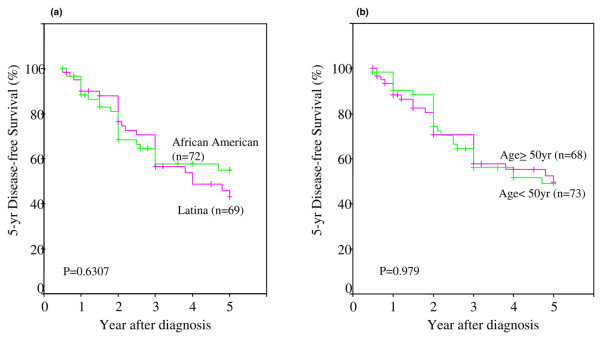
Five-year disease-free survival (DFS) in African-American and Latina women with breast cancer. **(a) **Kaplan-Meier survival curves comparing 5-year DFS between African-American (green) and Latina (fuchsia) women. **(b) **Kaplan-Meier survival curves comparing 5-year DFS in African-American and Latina women between less than 50 years of age (green) and above 50 years of age (fuchsia) at the time of diagnosis. The differences between the curves were estimated by log-rank test, and a *p *value of less than 0.05 was considered statistically significant.

The patients in this study were followed up for a mean of 4 years, and the time range of follow-up was 1 to 8 years. However, when the follow-up period exceeded 5 years, we counted and reported data only for the first 5 years. A recent review of cancer statistics confirms that African-American women with breast cancer have a shorter 5-year OS period [1] and premenopausal African-American women with breast cancer had a poorer prognosis than postmenopausal African-Americans [44]. However, the data on breast cancer prognosis in Latina women are limited. In our study, we had the distinct advantage of comparing the two ethnic groups who had similar socioeconomic status and access to care. In addition, there was no significant difference between our African-American and Latina patients with respect to their tumor pathology and stage of disease at the time of diagnosis. Results from Kaplan-Meier survival analysis from Figure 5a confirm that there is no significant difference in 5-year DFS between African-American and Latina women with breast cancer. However, both groups had a poor 5-year DFS rate. Next, we examined whether the age at diagnosis had any influence on 5-year DFS. Our data showed no significant difference. This suggests that women in our study have similar 5-year DFS rates, irrespective of their age at diagnosis. The overall 5-year DFS rate was about 50%.

### Effect of HER2/neu status on 5-year disease-free survival

**Figure 6:**
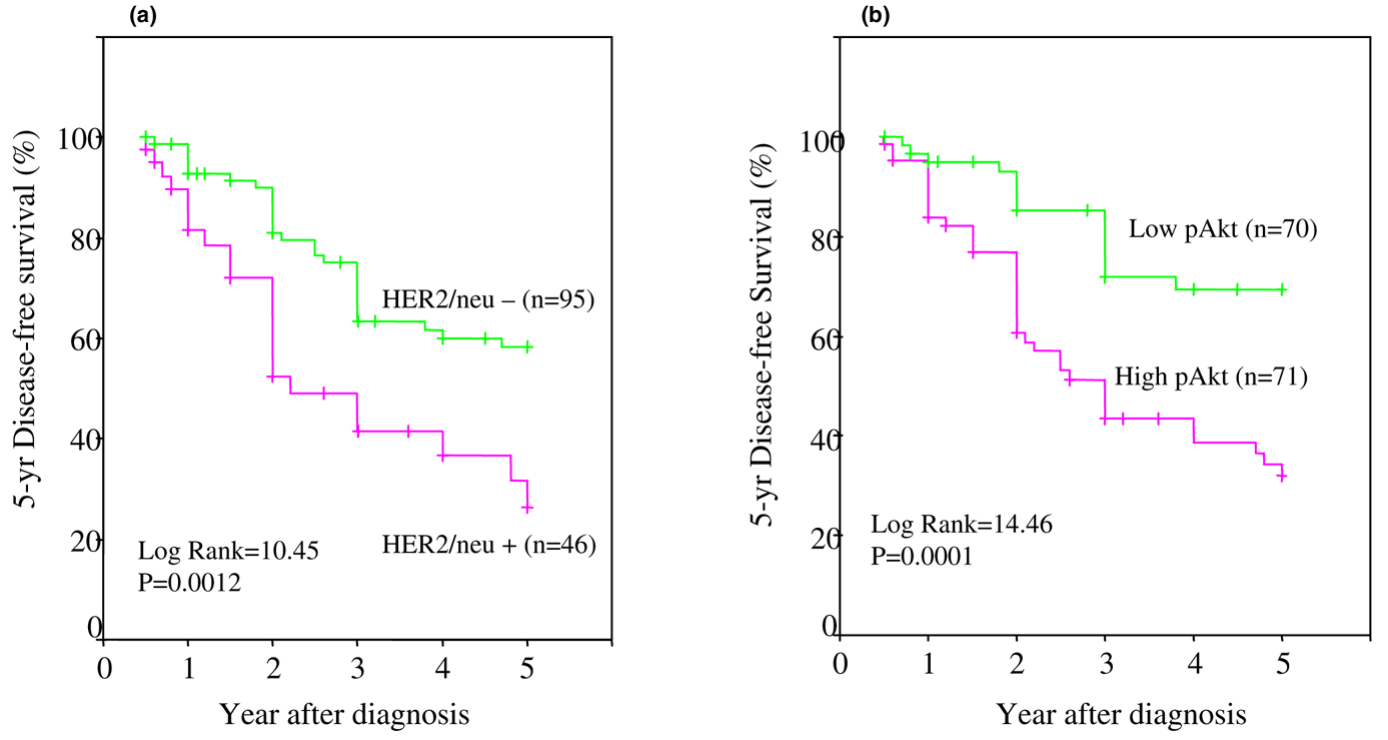
Five-year disease-free survival (DFS) in African-American and Latina women with HER2/neu or pAkt status. **(a) **Kaplan-Meier survival curves were used to compare the 5-year DFS between HER2/neu-positive (fuchsia) and HER2/neu-negative (green) tumors. **(b) **A similar comparison between high pAkt (fuchsia) and low pAkt (green) tumors in African-American and Latina women. The differences between the curves were estimated by log-rank test, and a *p *value of less than 0.05 was considered statistically significant.

We found that the probability of 5-year DFS was reduced significantly in patients with HER2/neu-positive tumors (Figure 6a). The median DFS time was 3.9 years for patients with HER2/neu-negative tumors but only 2.8 years for those with HER2/neu-positive tumors. As stated in Materials and methods, HER2/neu status was measured by IHC methods and information was obtained from clinical pathology charts.

### Effect of pAkt on 5-year disease-free survival

**Figure 7:**
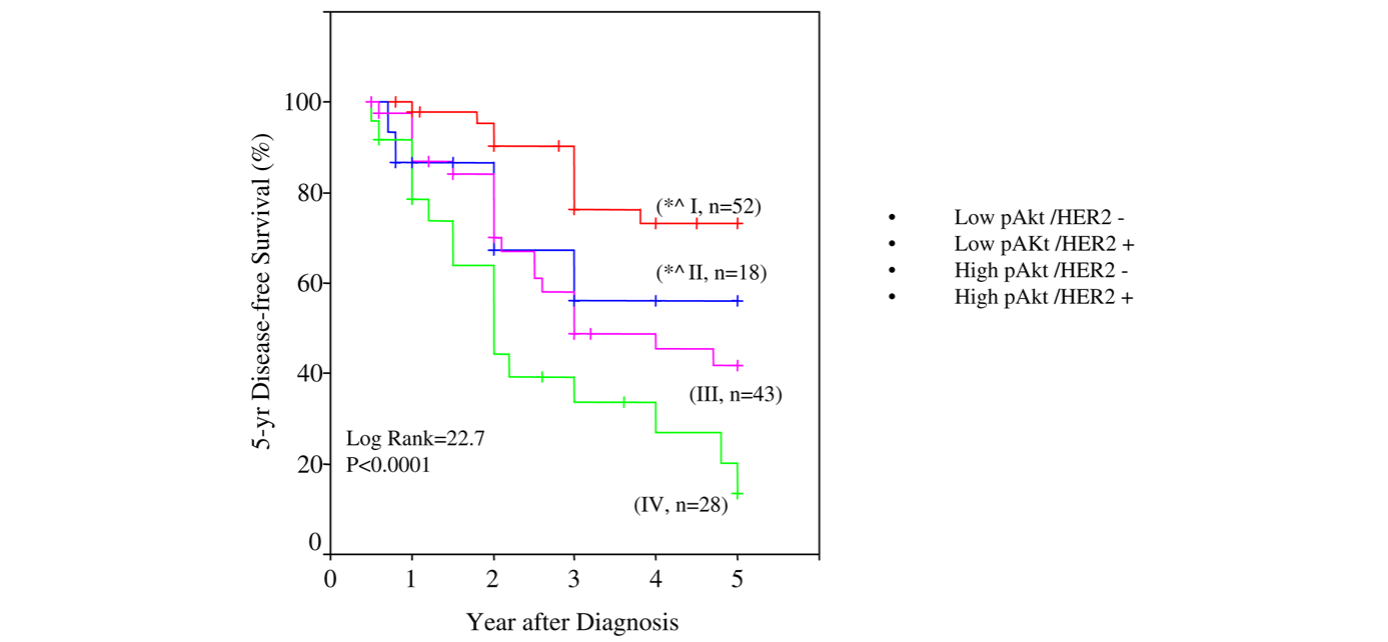
Five-year disease-free survival (DFS) in African-American and Latina patients with either high or low pAkt together with their HER2/neu status. Kaplan-Meier survival curves were used to compare the 5-year DFS among patients with low pAkt/HER2^- ^(I, red), low pAkt/HER2^+ ^(II, blue), high pAkt/HER2^- ^(III, fuchsia), and high pAkt/HER2^+ ^(IV, green). The differences between the curves were estimated by log-rank test, and a *p *value of less than 0.05 was considered statistically significant. *p < 0.005, the indicated groups compared to group IV (high pAkt/HER2^+^). ^*p *= 0.03, between the indicated two groups.

Table 3 had demonstrated that pAkt was significantly associated with an increase in HER2/neu-overexpressing breast tumors. We then examined whether the pAkt status had any influence on 5-year DFS and whether this association was directly linked to the HER/2neu status. Figure 6b shows that 5-year DFS was significantly reduced in patients with high pAkt compared with those with low pAkt. In comparison to patients with HER2/neu-negative tumors and with low pAkt levels, we observed the following: (a) HER2/neu overexpression led to a significant decrease in 5-year DFS, even in the presence of low pAkt; (b) high pAkt expression in the presence of HER2/neu-negative tumors led to a poorer 5-year DFS; and (c) those with both high pAkt and high HER2/neu tissue expression had the worst outcome, with a 5-year DFS rate of only 10% to 15% (Figure 7). The differences were highly significant at a *p *value of less than 0.0001 and with a log-rank value of 22.7.

Next, we evaluated the distant metastases and local recurrence rates within 5 years in these patients with different HER2/neu status and pAkt levels. Table 4 shows this evaluation. Overall, patients included in this study had 44% distant metastases and 7% local recurrence within 5 years. The frequency of distant metastases was significantly higher in patients with high pAkt group, regardless of their tumor HER2/neu status (Table 4). Patients whose breast tumors had both high HER2/neu and high pAkt1 (61.9%) had the highest association with distant metastases. Local recurrence was highest in patients with HER2/neu-positive tumors, independent of pAkt. However, these associations with local recurrence were not statistically significant, due to a lower number of patients with recurrence.

**Table 4:** pAkt level in relation to distant metastases and local recurrence

	Distant metastases		*P *value	Local recurrence		*P *value
	Yes (%)	No (%)		Yes (%)	No (%)	
HER2^+^/High pAkt	61.9	38.1	0.007	12.5	87.5	0.259
HER2^+^/Low pAkt	21.4	78.6		18.2	81.8	
HER2^-^/High pAkt	56.3	43.7		7.1	92.9	
HER2^-^/Low pAkt	31.3	68.7		0	100	

HER2^+^, HER2/neu 3+ determined by immunohistochemistry (IHC); HER2^-^, HER2/neu 2+ and 1+ and negative determined by IHC; High pAkt, pAkt level above median level (>36); Low pAkt, pAkt level of less than or equal to 36.

Since an increase in pAkt together with high HER2/neu expression was associated with tumor metastasis, we examined which factors would contribute most significantly to a reduction in 5-year DFS. We performed Cox regression with multivariate analysis to identify the RR of reducing 5-year DFS. Table 5 demonstrates that the following factors contribute significantly to a decrease in DFS: larger tumor size, positive lymph nodes, ER/PR-negative tumors, and HER2/neu-positive tumors. Most interestingly, in addition to these traditional risk factors, high pAkt was significantly associated (*p *< 0.006) with a decrease in 5-year DFS. Ethnicity (comparing African-American to Latina patients) and age (under or above 50 years) did not contribute to a decrease in 5-year DFS (Table 5).

**Table 5:** Estimation of relative risk of reduction of disease-free survival in African-American and Latina women with breast cancer using multivariate analysis

	Relative risk	95% CI	*P *value
Tumor size (≥ 5 cm versus <5 cm)	2.1	1.4–4.0	0.03
Lymph node (positive versus negative)	2.2	1.0–4.8	0.05
ER/PR status (negative versus positive)	1.9	1.0–3.3	0.04
pAkt status (high versus low)	2.5	1.3–4.8	0.006
HER2/neu status (positive versus negative)	1.7	1.0–3.3	0.05
Age (<50 years versus ≥ 50 years)	0.9	0.5–1.7	0.54
Ethnicity (African-American versus Latina)	1.2	0.7–2.2	0.54

CI, confidence interval; ER/PR, estrogen receptor/progesterone receptor; HER2/neu positive, HER2/neu 3+ examined by immunohistochemistry (IHC); HER2/neu negative, HER2/neu 2+ and HER2/neu 1+/negative by IHC; pAkt high, the pAkt index level above median level (>36); pAkt low, the pAkt index level less than or equal to median level (≤36).

### pAkt in tumor subtypes and influence on 5-year disease-free survival

Recent studies have suggested that breast cancer patients with certain tumor subtypes may be more resistant to therapy and therefore show a decrease in DFS and OS. Given that pAkt overexpression is associated with a decrease in DFS, we then asked whether it is possible that certain tumor subtypes, classified according to their receptor status, would be more likely to overexpress pAkt and whether these subtypes may subsequently have a shorter DFS. Hence, we examined the effect of high and low pAkt on the following tumor subtypes: (a) luminal A (ER/PR^+ ^and HER2^-^), (b) luminal B (ER/PR^+ ^and HER2^+^), (c) ER/PR^-^/HER2^+^, and (d) basal-like (triple-negative, ER/PR, and HER2^-^).

Our data in Table 6 show the levels of pAkt (median pAkt index) in different tumor subtypes: luminal A (ER/PR and HER2^-^), 13.4; luminal B (ER/PR^+ ^and HER2^+^), 42.3; ER/PR^- ^and HER2^+^, 56.3; and basal-like (ER/PR^- ^and HER2^-^), 35. These values suggest that pAkt levels are highest in ER/PR^- ^and HER2^+ ^tumors, followed by luminal B (ER/PR^+ ^and HER2^+^), and basal-like (ER/PR^- ^and HER2^-^) and are least in luminal A (ER/PR^+ ^and HER2^-^). In comparison to luminal A type, the levels of pAkt were higher in luminal B and ER/PR^-^/HER2^+ ^with statistical significance at a *p *value of less than or equal to 0.005, whereas the basal-like group was significant at a *p *value of 0.02.

**Table 6:** pAkt level in different subtypes of breast cancer

Subtype of breast cancer	Number	pAkt index (median)	*P *value
Luminal A (ER/PR^+ ^and HER2^-^)	38	13.4	
Luminal B (ER/PR^+ ^and HER2^+^)	44	42.3	0.005
ER/PR^- ^and HER2^+^	33	56.0	0.002
Basal-like (ER/PR^- ^and HER2^-^)	26	35.0	0.02

*P *values were compared with the luminal A group. ER/PR, estrogen receptor/progesterone receptor.

**Figure 8:**
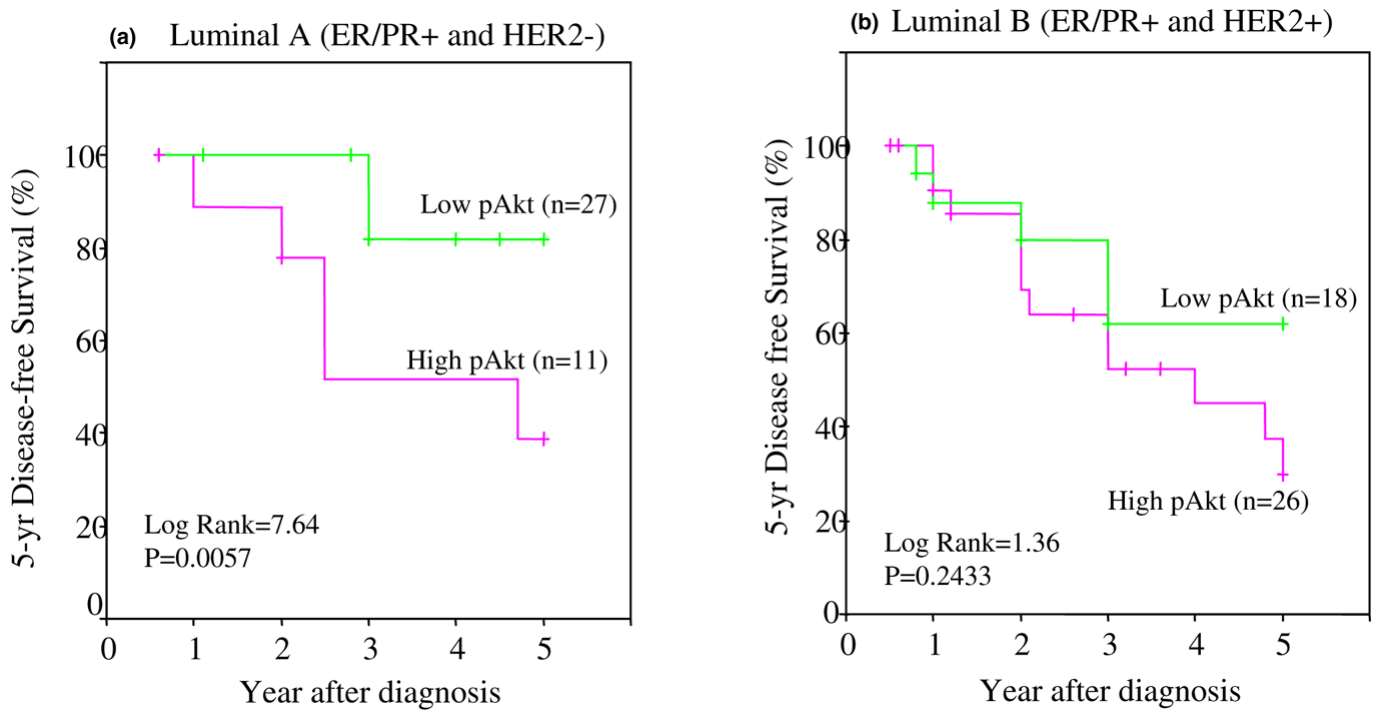
Five-year disease-free survival (DFS) in African-American and Latina women with luminal A and luminal B subtypes of breast cancer versus different levels of pAkt. Kaplan-Meier survival curves were used to compare the 5-year DFS between patients with high pAkt (fuchsia) and low pAkt (green) and with luminal A type of tumor **(a) **and luminal B type of tumor **(b)**. The differences between the curves were estimated by log-rank test, and a *p *value of less than or equal to 0.05 was considered statistically significant. ER, estrogen receptor; PR, progesterone receptor.

**Figure 9:**
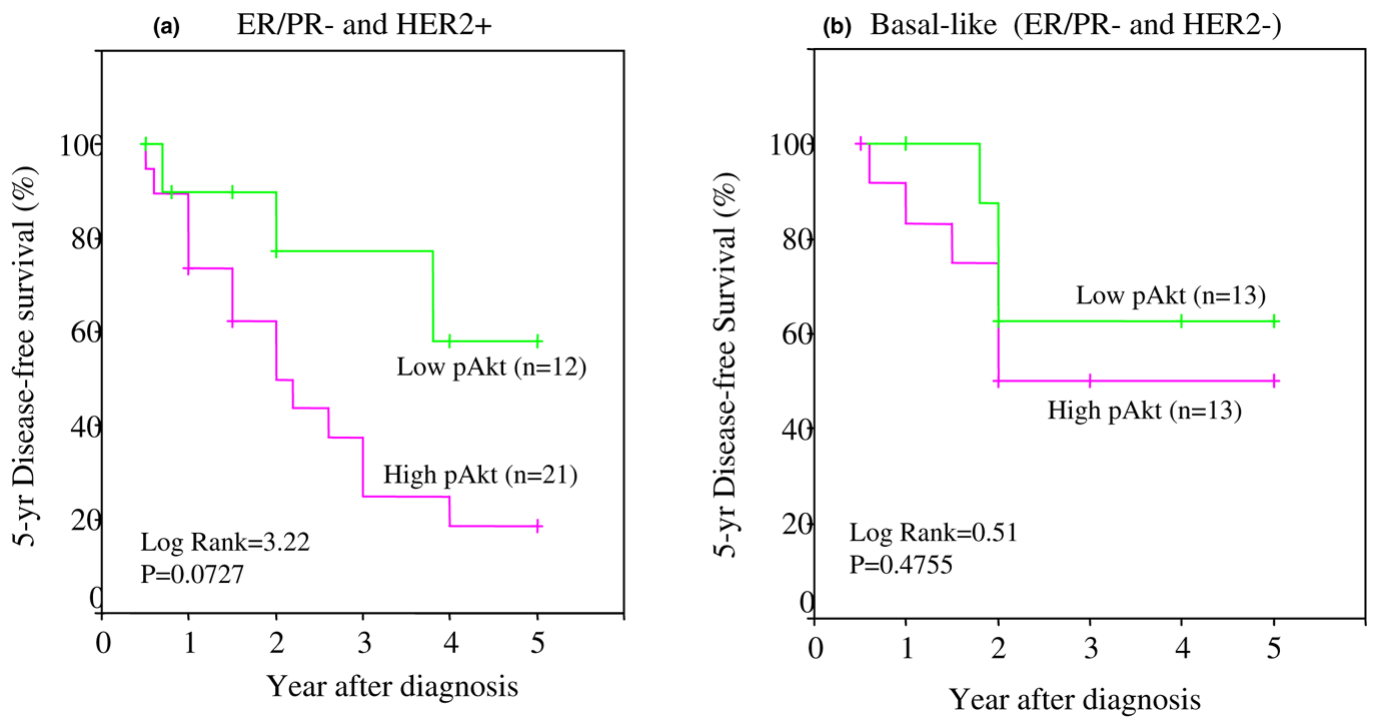
Five-year disease-free survival (DFS) in African-American and Latina women with ER/PR^- ^and HER2^+ ^and basal-like (ER/PR^-^HER2^-^) subtypes of breast cancer versus different levels of pAkt. Kaplan-Meier survival curves were used to compare the 5-year DFS between patients with high pAkt (fuchsia) and low pAkt (green) and with ER/PR^- ^and HER2^+ ^tumor **(a) **and basal-like (ER/PR^-^/HER2^-^) tumor **(b)**. The differences between the curves were estimated by log-rank test, and a *p *value of less than or equal to 0.05 was considered statistically significant. ER, estrogen receptor; PR, progesterone receptor.

Next, we examined the relationship between 5-year DFS and low and high pAkt levels in patients classified under different tumor subtypes. Figure 8a (luminal A, ER/PR^-^, and HER2^+^) shows a significant difference in DFS between low and high pAkt (*p *= 0.0057 and log-rank = 7.64). Tumors with high pAkt had poor DFS. The luminal B subtype (ER/PR^+ ^and HER2^+^) in Figure 8b showed poor DFS with both low and high pAkt levels. Although the difference was not statistically significant between the low and high pAkt groups, the overall DFS was considerably lower in tumors with high pAkt. Figure 9a shows ER/PR^- ^with HER2^+ ^subtype: In this group, patients with high pAkt had a very poor 5-year DFS (only 20%). In contrast, those with low pAkt had a DFS of almost 60%. The basal-like tumors (ER/PR^- ^with HER2^-^) in Figure 9b showed poor but similar DFS rates between low and high pAkt levels. Hence, the difference was not statistically significant.

**Figure 10:**
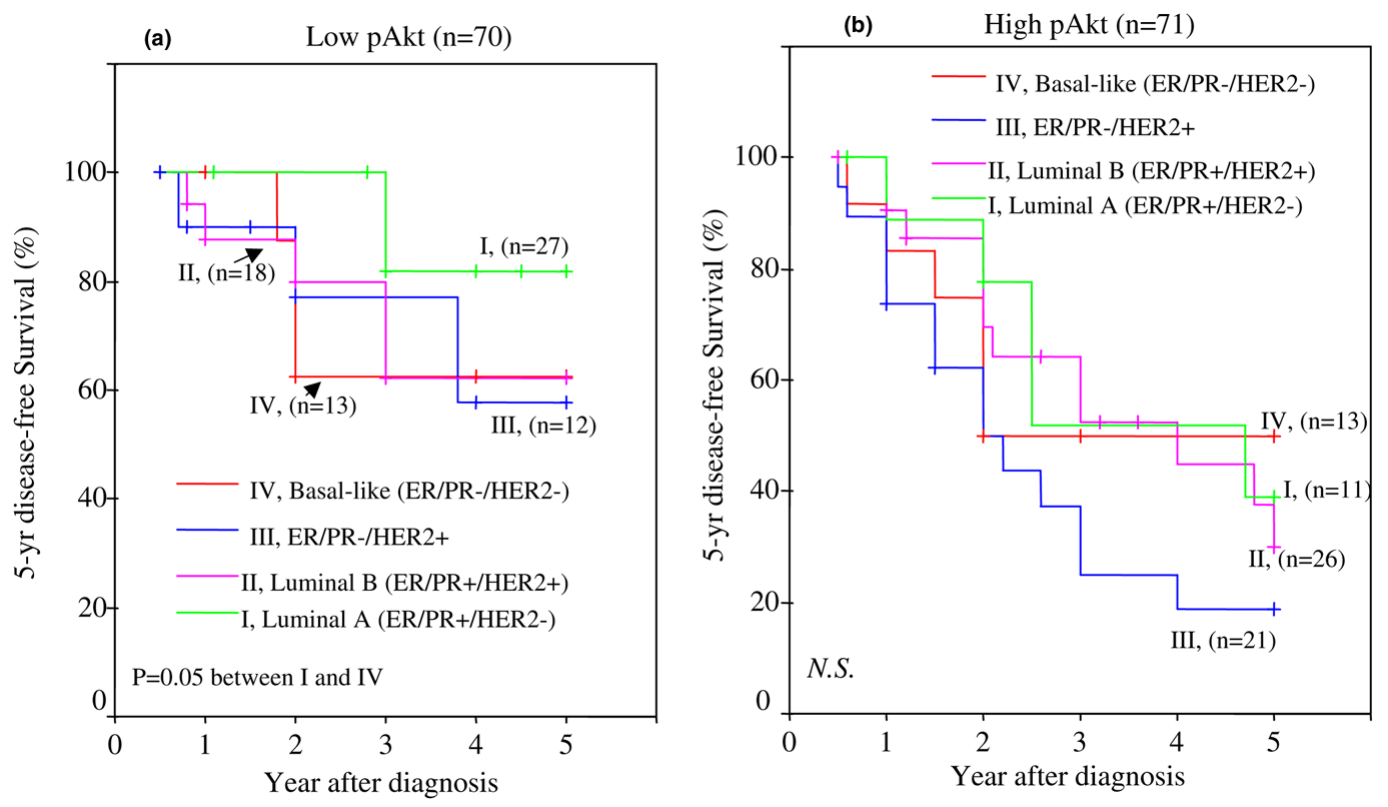
Five-year disease-free survival (DFS) in African-American and Latina women with different levels of pAkt versus different types of breast cancer. Patients were grouped as low pAkt (median level of pAkt index of less than or equal to 36) **(a) **and high pAkt (median level of pAkt index of greater than 36) **(b)**. Then Kaplan-Meier survival curves were used to compare the 5-year DFS between patients with different subtypes of tumors: I, luminal A type of tumor (green); II, luminal B type of tumor (fuchsia); III, ER/PR^- ^and HER2^+ ^tumor (blue); and IV, basal-like type of tumor (red). The differences between the curves were estimated by log-rank test, and a *p *value of less than or equal to 0.05 was considered statistically significant. ER, estrogen receptor; N.S., no statistical significance between the curves; PR, progesterone receptor.

Next, we compared differences in DFS between the different tumor subtypes and within the high or low pAkt groups. Figure 10a shows the differences within the low pAkt group. The data suggest that, in comparison to the luminal A subtype (ER/PR^+^/HER2^-^), the 5-year DFS rate was lower in luminal B (ER/PR^+^/HER2^+^), ER/PR^-^/HER2^+^, and basal-like (ER/PR^-^/HER2^-^). The difference was statistically significant (*p *= 0.05) with the basal-like group. These observations demonstrate that basal-like tumors have the worse DFS, despite low levels of tissue pAkt. Next, when we compared the DFS rates among the patients with different tumor subtypes with high pAkt, we observed that all four subtypes had a very poor DFS rate (Figure 10b). However, the ER/PR^-^/HER2^+ ^subtype had the worse outcome.

## Discussion

Among the three human isoforms Akt1, Akt2, and Akt3, it has been suggested that Akt1 may have an important role in tumor initiation and tumor growth [45-48]. Breast cancer patients with high-grade tumors and late stage of diagnosis had activated Akt1 in their tumor cells and their disease had a poor outcome [49]. Furthermore, patients who had a poor outcome to endocrine therapy also had activated Akt1 in their tumor cells [32]. Akt2 frequently has been found to be upregulated in HER2/neu-positive breast tumors and may contribute to tumor aggressiveness and metastases [48,50,51]. Akt3 expression is exclusive to more advance and hormone-independent breast cancer cells [52]. In the present study, we used phosphor-Akt (Ser473) antibody, which detects Akt1 only when phosphorylated at serine 473 and which detects Akt2 and Akt3 only when phosphorylated at equivalent sites. Therefore, the activated pAkt in breast tumors in our patient cohort determined by IHC may not be limited to phosphorylation of Akt1 or Akt2 only. A study in 280 postmenopausal Swedish women with breast cancer indicated that expression of pAkt was significantly associated with both Akt1 and Akt2 staining; however, the correlation was stronger for Akt1 than for Akt2 [37]. Another study in 402 ERα-positive breast cancer patients [36] showed clearly that cytoplasmic Akt1 and Akt3, but not Akt2, expression was correlated significantly with pAkt expression. We have also examined the association of pAkt in relation to Akt1 and Akt2 in our patient cohort. The mRNA levels of Akt1 and Akt2 were determined by quantitative real-time polymerase chain reaction in 60 samples. The results indicate that the pAkt protein expression was more associated with Akt1, and there was a significant correlation between Akt1 and Akt2 at the mRNA level (data not shown). Our results with different Akt isoforms are more in agreement with the Swedish study [37]. Studies with both Japanese and Swedish women with breast cancer indicated that activation of pAkt in breast cancer was more likely to be associated with ER/PR-negative tumors [34,37].

The PI3K/Akt signaling pathway is known to promote growth factor-mediated cell growth, proliferation, migration, and survival [53-57]. Activation of the PI3K/Akt signaling pathway by HER2/neu or other growth factors has been suggested to contribute to multidrug resistance in breast cancer [10,57]. In addition, the role of the PI3K/Akt pathway has been investigated in many different types of tumors, including oral, head and neck, and squamous cell tumors. All of these tumors are associated with poor prognosis [58-60]. Activation of Akt at serine 473 has been reported to induce resistance to chemotherapy and to tamoxifen treatment in Caucasian and Asian women with breast cancer [34-36]. Recently, the activation of Akt has been shown in Japanese as well as Caucasian women with HER2/neu-positive breast cancer [34,37,61]. Consequently, activation of Akt downstream of HER2/neu receptor may play a key role in the development of resistance to chemotherapy and to anti-HER2/neu receptor antibody, trastuzumab, in HER2/neu-overexpressing breast tumors.

Our study was designed to understand the interactions between activation of tissue Akt with and without corresponding overexpression of HER2/neu for assessment of disease outcome in African-American and Latina women with breast cancer. Similar to the data on Caucasian and Japanese women [34,37,61], the expression of pAkt in breast tumors from African-American and Latina women in our study was significantly associated with HER2/neu-positive tumors. As mentioned in the Introduction and in Materials and methods, our medical center is located in South Central Los Angeles and serves primarily underserved populations, mainly African-American and Latina patients. Since the population of Caucasians served is less than 2%, we have not included this group in our study.

The numbers of African-American and Latina patients recruited in our study were fairly similar, and the two groups had similar socioeconomic status and access to care. Hence, we feel confident that our study minimizes possible discrepancies due to non-biological factors. The distribution of patients below or above 50 years of age was fairly similar in our cohort. With respect to tumor histopathology, there was a significant difference (*p *= 0.001 to 0.004) with the following parameters: a larger number of tumors were less than 5 cm, whereas a greater number had positive lymph node metastasis (63.1%), AJCC stage between 0 and II (62.4%), and poorly differentiated tumors (69.5%), and 32.6% had HER2/neu-positive tumors. Fifty-seven point four percent had ER/PR^+ ^status whereas 42.6% had ER/PR^- ^status. This difference was not significant (*p *= 0.086).

Overall, there was no statistical difference in the pAkt levels in tumor tissues between African-American and Latina patients. Similarly, there was no statistical difference in the pAkt levels between patients under or above 50 years of age, with tumors smaller or larger than 5 cm, between AJCC stage below or above stage II, and those with ER/PR^+ ^or ER/PR^- ^tumors. In contrast, pAkt levels were significantly different in tumor tissues obtained from patients with lymph node metastasis and those with HER2/neu-positive tumors. Patients with poorly differentiated tumors also had higher pAkt levels, although the difference was not highly significant (*p *= 0.094). Similarly, the level of pAkt in patients with ER/PR-negative tumors was higher than in patients with ER/PR-positive tumors; however, this difference was not statistically significant (Table 3). Hence, our data confirm that pAkt is elevated significantly only in tumor tissues from patients with lymph node metastasis, HER2/neu overexpression, and possibly those with ER/PR-negative tumors.

As we examined the association between HER2/neu overexpression and increase in pAkt in our breast cancer patients, we discovered some unique features. First, more than 70% of patients with HER2/neu-positive tumors had overexpression of pAkt, and the high pAkt tumors were associated with positive lymph nodes. Although a similar study on women in Germany showed that 71% of pAkt-positive tumors were HER2/neu-positive, they had included both HER2/neu 2+ and 3+ positive patients. In addition, their study was conducted in patients with node-negative breast cancer [61]. The studies on Japanese and Swedish women [34,37] showed that 43% to 44% of HER2/neu-positive tumors had a corresponding increase in pAkt expression. The HER2/neu-positive tumors in the Japanese group included HER2/neu 2+ and 3+ as determined by IHC, and for Swedish group, the positive HER2/neu was determined using a FACSCalibur Flow Cytometer (BD Biosciences, San Jose, CA, USA). In comparison, our study showed that more than 70% of African-American and Latina women with HER2/neu 3+ positive tumors had overexpression of pAkt in their tumors at the time of diagnosis. This is the highest degree of pAkt overexpression reported for any ethnic group. This observation leads us to speculate that pAkt overexpression in breast tumors from African-American and Latina women may be one important factor contributing to their overall decrease in DFS. Our data also demonstrate for the first time that patients with high pAkt but with HER2/neu-negative tumors also had poor 5-year DFS. Furthermore, multivariate analysis adjusted for traditional prognostic indicators such as lymph node involvement, tumor size, HER2/neu status, and ER/PR status demonstrated that pAkt level alone is a stronger predictor for a decrease in DFS.

Recent studies suggested that a high frequency of basal-like (ER/PR^-^/HER2^-^) breast cancer may contribute to the poor prognosis of young African-American women [44]. When we examined the 5-year DFS pattern with respect to pAkt levels in connection with HER2/neu status and with tumor subtypes, we discovered an interesting role for pAkt. In comparison to patients with HER2/neu-negative tumors and with low pAkt levels, we observed the following: (a) HER2/neu overexpression led to a significant decrease in 5-year DFS, even in the presence of low pAkt; (b) high pAkt expression in the presence of HER2/neu-negative tumors had poorer 5-year DFS; and (c) those with both high pAkt and high HER2/neu tissue expression had the worst outcome, with a 5-year DFS rate of only 10% to 15%. The differences were highly significant at a *p *value of less than 0.0001 and with a log-rank value of 22.7.

When we examined the association of pAkt with distant metastasis and local recurrence, we observed the following: In general, patients in our study had 44% distant metastases and 7% local recurrence within 5 years. We were surprised at such a low local recurrence rate. Interestingly, however, the frequency of distant metastases was significantly high in patients with high pAkt regardless of their tumor HER2/neu status. Patients whose breast tumors had both high HER2/neu and high pAkt had the highest association with distant metastases (61.9%). Local recurrence was highest in patients with HER2/neu-positive tumors, independent of pAkt. We believe that, in our current study, the association with local recurrence was not statistically significant, due to a smaller sample size of patients with local recurrence.

Next, we examined what factors would contribute to a decrease in 5-year DFS. Using Cox regression with multivariate analysis, we determined that the following contributed significantly: larger tumor size, positive lymph nodes, ER/PR-negative tumors, and HER2/neu-positive tumors. Most interestingly, in addition to these traditional risk factors, high pAkt was significantly associated (*p *< 0.006) with a decrease in 5-year DFS. Ethnicity (comparing African-American to Latina patients) and age (under or above 50 years) did not contribute to a decrease in 5-year DFS. Hence, between the African-American and Latina populations, ethnicity or race is not a significant contributor of poor DFS.

Perou and colleagues [62] and Sørlie and colleagues [63] elegantly demonstrated that breast carcinomas can also be subdivided based on gene expression analysis. However, it is unclear whether the DFS and OS rates in these different subtypes can be influenced by overexpression of pAkt. Given that pAkt overexpression is associated with a decrease in DFS, we then asked whether it is possible that certain tumor subtypes, classified according to their receptor status, would be more likely to overexpress pAkt and whether these subtypes may subsequently have a shorter DFS. We examined the effect of high and low pAkt on the following tumor subtypes: (a) luminal A (ER/PR^+ ^and HER2^-^), (b) luminal B (ER/PR^+ ^and HER2^+^), (c) ER/PR^-^/HER2^+^, and (d) basal-like (triple-negative, ER/PR, and HER2^-^). First, our data revealed that pAkt levels were highest in ER/PR^- ^and HER2^+ ^tumors, followed by luminal B (ER/PR+ and HER2+), and basal-like (ER/PR^- ^and HER2^-^) and least in luminal A (ER/PR+ and HER2^-^). In comparison with luminal A type, the levels of pAkt were higher in luminal B and ER/PR^-^/HER2^+ ^subtypes with statistical significance at a *p *value of less than or equal to 0.005. The basal-like group was significant at a *p *value of 0.02. Second, we examined the relationship between 5-year DFS and low and high pAkt levels in patients classified under different tumor subtypes. Luminal A tumors (ER/PR^- ^and HER2^+^) showed a significant difference in DFS between low and high pAkt (*p *= 0.0057 and log-rank = 7.64). Tumors with high pAkt had poor DFS. Luminal B subtype (ER/PR^+ ^and HER2^+^) also showed poor DFS with both low and high pAkt levels. Although the difference was not statistically significant between the low and high pAkt groups, the overall DFS was considerably lower in tumors with high pAkt. In the ER/PR^-^/HER2^+ ^subtype, patients with high pAkt had a very poor 5-year DFS (only 20%). In contrast, those with low pAkt had a DFS of almost 60%. The basal-like tumors (ER/PR^- ^with HER2^-^) showed poor but similar DFS rates between low and high pAkt levels. Hence, the difference was not statistically significant. Finally, we compared differences in DFS between the different tumor subtypes, but within the high or low pAkt group. Our data suggest that, in comparison with the luminal A subtype (ER/PR^+^/HER2^-^), the 5-year DFS rate was significantly lower in luminal B (ER/PR^+^/HER2^+^), ER/PR^-^/HER2^+^, and basal-like (ER/PR^-^/HER2^-^). However, the difference was statistically significant (*p *= 0.05) with the basal-like group. These observations demonstrate that basal-like tumors have the worse DFS, despite low levels of tissue pAkt. Next, when we compared the DFS rates among the patients with different tumor subtypes with high pAkt, we observed that all four subtypes had a very poor DFS rate. However, the ER/PR^-^/HER2^+ ^subtype had the worse outcome.

## Conclusion

Our studies confirm that an increase in tissue pAkt expression in HER2/neu-overexpressing breast cancer patients leads to poor outcome with a significant decrease in 5-year DFS. We also show that an increase in pAkt in HER2/neu-negative patients leads to poor DFS. In addition to established prognostic markers such as nodal involvement, tumor size, ER/PR status, and HER2/neu status, pAkt is an independent and strong predictor for a decrease in DFS. Therefore, pAkt may prove to be an important therapeutic target. A unique feature of our study is that more than 70% of African-American and Latina women with HER2/neu-overexpressing breast tumors are likely to have an increase in their tissue pAkt levels. This feature, however, needs to be validated using a larger cohort of minority patients and be compared to Caucasian patients with similar socioeconomic status, access to care, and stage of disease at diagnosis. Several comparative studies between African-American and Caucasian groups have confirmed significant differences in survival rates between the two groups. African-Americans tend to have poorer outcome [64-66]. It will be an important opportunity to target pAkt for therapy in these high-risk populations. Overexpression of pAkt may be a powerful prognostic marker for DFS in breast cancer.

Additional studies are required to understand the role of pAkt in the development of drug resistance. In this study, we were not able to ascertain the role of pAkt in HER2/neu-overexpressing breast tumors resistant to trastuzumab. This is because the patients who had received trastuzumab prior to 2004 were very few. Further studies are needed to determine whether the level of pAkt contributes to resistance to trastuzumab in these minority women with HER2/neu-positive breast cancer. Biological or genetic differences resulting from mutations in the PI3K pathway could account for the activated Akt tumors, in addition to mutations or loss of PTEN (phosphatase and tensin homolog deleted on chromosome 10) in HER2/neu-overexpressing breast tumors. Any of these factors could contribute to a poor response to therapy. More studies with a larger sample size will improve our understanding of the role of pAkt and poor disease outcome in all patients with breast cancer.

## Abbreviations

AJCC = American Joint Committee on Cancer; ATCC = American Type Culture Collection (Manassas, VA, USA); DFS = disease-free survival; EGFR = epidermal growth factor receptor; ER = estrogen receptor; IHC = immunohistochemistry; OS = overall survival; PI3K = phosphatidylinositol-3-kinase; PR = progesterone receptor; RR = relative risk; SD = standard deviation.

## Competing interests

The authors declare that they have no competing interests.

## Authors' contributions

YW and JVV were responsible for data collection, analysis, manuscript preparation, and editing. HM, RC, IA, and SC provided the clinical support for patient recruitment and were involved in the study design. DS provided overall guidance, direction, and critical review of the study design. All authors read and approved the final manuscript.
